# Rare deleterious variants of *NOTCH1*, *GATA4*, *SMAD6*, and *ROBO4* are enriched in BAV with early onset complications but not in BAV with heritable thoracic aortic disease

**DOI:** 10.1002/mgg3.1406

**Published:** 2020-08-03

**Authors:** Fadi I. Musfee, Dongchuan Guo, Amélie C. Pinard, Ellen M. Hostetler, Elizabeth E. Blue, Deborah A. Nickerson, University of Washington Center for Mendelian Genomics (UW‐CMG), Michael J. Bamshad, Dianna M. Milewicz, Siddharth K. Prakash

**Affiliations:** ^1^ Department of Epidemiology School of Public Health University of Texas Health Science Center at Houston Houston TX USA; ^2^ Department of Internal Medicine McGovern Medical School University of Texas Health Science Center at Houston Houston TX USA; ^3^ Department of Medicine University of Washington School of Medicine Seattle WA USA; ^4^ Genome Sciences University of Washington School of Medicine Seattle WA USA; ^5^ Department of Pediatrics University of Washington School of Medicine Seattle WA USA

**Keywords:** bicuspid aortic valve, thoracic aortic aneurysm, whole exome sequence

## Abstract

**Background:**

Bicuspid aortic valve (BAV) is the most common cardiovascular malformation in adults, with a prevalence of 0.5%–2%. The prevalence of BAV in cohorts who were ascertained due to thoracic aortic aneurysms or acute aortic dissections (TAD) is as high as 20%. However, the contribution of causal BAV genes to TAD is not known. Therefore, we evaluated rare deleterious variants of *GATA4*, *NOTCH1*, *SMAD6*, or *ROBO4* in patients with BAV who presented with TAD.

**Methods:**

Our cohort consisted of 487 probands with Heritable Thoracic Aortic Aneurysms or Dissections (HTAD, 12% BAV, 29% female) and 63 probands with Early onset complications of Bicuspid Aortic Valve disease (EBAV, 63% TAD, 34% female). After whole exome sequencing, we functionally annotated *GATA4*, *NOTCH1*, *SMAD6*, and *ROBO4* variants and compared the prevalence of rare variants in these genes to controls without HTAD.

**Results:**

We identified 11 rare deleterious variants of *GATA4*, *SMAD6*, or *ROBO4* in 12 (18%) EBAV cases. The burden of rare *SMAD6* and *GATA4* variants was significantly enriched in EBAV but not in HTAD cases, even among HTAD cases with BAV (*p* < .003).

**Conclusion:**

Rare variants of *NOTCH1*, *ROBO4*, *SMAD6*, or *GATA4* do not significantly contribute to BAV in cohorts with HTAD. We conclude that BAV patients who present with HTAD are a genetically distinct subgroup with implications for genetic testing and prognosis.

## Graphical abstract

Bicuspid aortic valve (BAV) is the most common congenital heart defect in adults and is a frequent risk factor for heritable thoracic aortic aneurysms predisposing to acute aortic dissections (HTAD). To elucidate the roles of known causal BAV genes in HTAD, we determined if individuals who present with both BAV and HTAD harbor an increased burden of rare deleterious variants in NOTCH1, GATA4, SMAD6 or ROBO4. We found that variants of these genes do not significantly contribute to HTAD, even in cases with BAV. These results indicate that BAV patients who present due to complications of HTAD have markedly different genetic and clinical profiles than BAV patients who present due to valvular heart disease. Therefore, additional factors are probably required to drive clinically meaningful enlargement and dissection of aneurysms in BAV patients.

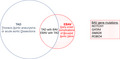

## 1 INTRODUCTION

Bicuspid aortic valve (BAV) is the most common congenital cardiovascular malformation in adults, with a prevalence of 0.5%–2% that is increased 10‐fold (5%–15%) in patients with heritable thoracic aortic aneurysms predisposing to acute aortic dissections (HTAD) (Foffa et al., 2013). BAV disease may occur as a feature of genetic syndromes, but primarily occurs without associated syndromic features (Gould et al., 2019). BAV is inherited as an autosomal dominant trait with high heritability (70%–90%) and is frequently associated with other left‐sided congenital lesions such as aortic coarctation (Cripe, Andelfinger, Martin, Shooner, & Benson, 2004). Pathogenic variants in *NOTCH1* (190198),* GATA4* (600576),* SMAD6* (602931) or *ROBO4* (607528) are the most commonly identified cause of familial non‐syndromic BAV (Foffa et al., 2013; Gould et al., 2019; Tan et al., 2012). To elucidate the roles of causal BAV genes in HTAD, we set out to determine if patients who present with both TAD and BAV harbor mutations in *NOTCH1*, *GATA4*, *SMAD6*, or *ROBO4*. We compared the burden of rare variants in two diverse cohorts who were either recruited primarily due to HTAD (HTAD cohort) with a significant proportion who also have BAV (12%), or due to early onset complications of BAV disease (EBAV cohort), primarily with concomitant TAD (63%). We hypothesized that rare deleterious variants of causal BAV genes may be enriched in cases with BAV and TAD in both cohorts.

## 2 MATERIALS AND METHODS

The study protocol was approved by the Committee for the Protection of Human Subjects at the University of Texas Health Science Center at Houston. We analyzed whole exome sequences of 487 European ancestry HTAD probands (12% BAV, 29% female, mean age 46 ± 9 years) and 63 European ancestry EBAV probands (35% female, 63% TAD, mean age 29 ± 12 years) according to our previously published methods (Zhang et al., 2018). EBAV subjects were selected due to early onset and severe complications of BAV, including TAD or valvular disease requiring intervention. The characteristics of the HTAD cohort, which was restricted to individuals who did not have syndromic features, were previously described (Guo et al., 2017; Kwartler et al., 2018). More than 70% of HTAD subjects were ascertained due to acute thoracic aortic dissections. We used ANNOVAR (v. April 16, 2018) for variant annotation and filtered the dataset to select heterozygous variants with minor allele frequencies (MAF) <0.5% in the Genome Aggregation Database (https://gnomad.broadinstitute.org) that are either stop‐gain, splice‐site, or missense mutations with scaled Combined Annotation Dependent Depletion (CADD) scores ≥20 (Wang, Li, & Hakonarson, 2010). We refer to these as rare deleterious variants. We used GEnome MINIng software (GeMINI v. 0.20) to select rare variants and visualized candidate variants using the Integrative Genome Viewer (IGV, v 2.3.97) (Paila, Chapman, Kirchner, Quinlan, & Gardner, 2013). All prioritized variants were validated using Sanger sequencing. BAV and TAD status was recorded for each genotyped individual by self‐reported questionnaires and was validated whenever possible by reviewing medical records. We compared the burden of rare *NOTCH1* (NG_007458.1), *SMAD6* (NG_012244.2), *ROBO4* (NM_019055.6), and *GATA4* (NG_008177.2) variants in HTAD probands with BAV and 4,300 unrelated European ancestry control sequences from the Exome Aggregation Consortium using Fisher's exact tests (Lek et al., 2016). We also assessed the familial segregation of rare variants in 101 first‐degree relatives of HTAD probands.

## 3 RESULTS

We identified 60 probands with HTAD who also had BAV (33% female, mean age at diagnosis 42 ± 9 years). Almost half of these patients had moderate or severe valvular disease and most had previous aortic or valvular surgical interventions (Table 1). Rare deleterious variants (CADD >20) of the four causal BAV genes were not significantly enriched in HTAD cases with BAV compared with European ancestry controls (Table 2). We identified 22 *NOTCH1* variants in 27 HTAD subjects (26 probands), 6 *SMAD6* variants in 15 HTAD subjects (14 probands), 11 *ROBO4* variants in 18 HTAD subjects (18 probands), and 4 GATA4 variants in 6 HTAD subjects (6 probands). However, only one subject with a *ROBO4* variant had BAV or a family history of BAV, and none of these variants segregated with HTAD or BAV in pedigrees with available data.

**Table 1:** Characteristics of study cohorts

Cohort	HTAD BAV *N* (%) or Mean (±SD)	EBAV *N* (%) or Mean (±SD)	*p* value HTAD BAV vs EBAV
	*N* = 60	*N* = 63	
Female	21 (35)	21 (33)	.846
Age (years)	42 (9)	29 (12)	<.0001
Aortic dissection	40 (67)	1 (2)	<.001
Aortic stenosis	4 (10)	16 (25)	.044
Aortic regurgitation	18 (45)	23 (37)	.391
Valve or aortic surgery	55 (92)	19 (30)	<.001
Rare deleterious variants	1 (14)	11 (65)	.034

Note

HTAD: probands with heritable thoracic aortic aneurysms or acute aortic dissections; BAV, bicuspid aortic valve; EBAV, proband with BAV who had early onset complications requiring intervention prior to age 30; Age: age when enrolled in the study; Rare deleterious variants: heterozygous variants of *NOTCH1*, *SMAD6*, *GATA4*, and *ROBO4* with minor allele frequencies (MAF) <0.5% in the Genome Aggregation Database that were either stop‐gain, splice‐site, or missense mutations with scaled Combined Annotation Dependent Depletion (CADD) scores ≥20. The percentage of all rare variants that meet these criteria is in parentheses.

**Table 2:** Burden analysis of *NOTCH1*,* SMAD6*, *ROBO4*, and *GATA4* variants

Gene	HTAD + BAV variants (*n* = 60)	*p*	EBAV variants (*n* = 63)	*p*	ESP_EA variants (*n* = 4,300)
NOTCH1	0	.163	1	.432	130
SMAD6	0	.727	4	.001	23
ROBO4	1	.572	3	.062	60
GATA4	0	.779	3	.003	18

Note

HTAD + BAV: Hereditable Thoracic Aortic Aneurysm probands with Bicuspid Aortic Valve; EBAV: Early onset complications of Bicuspid Aortic Valve disease; ESP_EA: Exome Sequencing Project_European Ancestry. *p* values were generated using one‐sided Fisher's exact tests. In total, we identified 40 variants of *NOTCH1*, *SMAD6*, *ROBO4*, or *GATA4* with MAF <0.5% and CADD ≥20 in the HTAD cohort, but only one variant, in *ROBO4*, was in a proband with BAV. EBAV variants are listed in the text. The complete list of HTAD rare variants: *NOTCH1* (NG_007458.1): p.Arg128His, p.Arg234His, p.Arg504His, p.Thr586Ile, p.Ala624Thr, p.Gly842del, p.Arg892His, p.Arg912Trp, p.Asn1023Ser, p.Cys1133Gly, p.Arg1279Cys, p.Pro1337Arg, p.Ala1343Val, p.Asp1439Asn, p.Thr1573Met, p.Arg1633His, p.Gln1691His, p.Trp1813Leu, p.Pro2122Leu, p.Arg2372Gln; *SMAD6* (NG_012244.2): Asp21Asn, p.Gly25_Gly26del, p.Pro47Ser, p.Gly97Glu, p.Cys121Tyr, p.Ser333Asn; *ROBO4* (NM_019055.6): c.1685+1 G>A, p.Arg129Pro, p.Ala213Thr, p.Arg492Gln, p.Leu698Met, p.Arg776Cys, p.Arg908Gln, p.Gly920Ser, p.Val929Ile, p.Pro952Thr, p.Arg995His; *GATA4* (NG_008177.2): p.Glu107Asp, p.Ala176_Ala177del, p.Asp185Tyr, p.Ser337_Glu338del.

In the EBAV cohort, the prevalence of TAD (63%), aortic dissection (2%), aortic valve stenosis (25%) or regurgitation (35%) and any aortic surgical intervention (25%) was similar to published data (Table 1). We identified 11 rare deleterious variants of *NOTCH1*,* GATA4*,* SMAD6*, or *ROBO4* in 12 EBAV subjects: 1 *NOTCH1* variant: p.Gly936Ser; 4 *SMAD6* variants: p.Arg381fs, p.Asp359fs, p.Gly29Ala, and p.Leu191Pro; 3 *ROBO4* variants: p.Glu797*, p.Val235Met, and p.Asn707Lys; and 3 *GATA4* variants: p.Glu314Asp, p.Ala412Val, and an inherited genomic duplication of the *GATA4* locus on chromosome 8 (hg19 coordinates: 11,403,560‐11,853,760). Burden tests showed that rare variants of *SMAD6* and *GATA4* are significantly enriched in EBAV cases in comparison with controls (Table 2). Rare *NOTCH1* and *ROBO4* variants were also enriched in the EBAV cohort, but this was not statistically significant due to limited power in this small data set.

The diagnosis of BAV in patients who present primarily due to TAD, or the diagnosis of TAD in patients who present primarily due to BAV, may be underestimated due to ascertainment bias. We tried to minimize this issue by reviewing operative reports and aortic images of all rare variant carriers. The statistical power of burden testing was limited by the small sample size and by confounding due to comparisons with unselected database controls. The sample size was also insufficient to correlate differences in BAV or aortic phenotypes with the mutated gene. Nevertheless, we were able to confirm the enrichment of causal BAV genes in the much smaller EBAV cohort.

## 4 CONCLUSION

We did not find support for the relationship between rare coding variation of *NOTCH1*, *SMAD6*, *GATA4*, and *ROBO4* and HTAD, even in cases with BAV. Our results indicate that BAV patients who present due to complications of HTAD may have distinct genetic and clinical profiles from BAV patients who present due to valvular heart disease. Therefore, additional factors are probably required to drive clinically meaningful enlargement and dissection of aneurysms in BAV patients.

## CONFLICT OF INTEREST

The authors have no conflicts of interest to disclose.

## AUTHOR CONTRIBUTIONS

Fadi Musfee contributed to formal analysis, writing—original draft. Dongchuan Guo, Amélie Pinard, and Ellen Hostetler contributed to data curation, methodology. Elizabeth Blue, Deborah Nickerson, and Michael Bamshad contributed to methodology, software, and project administration. Dianna Milewicz and Siddharth Prakash contributed to conceptualization, funding acquisition, writing—review and editing.

## Data Availability

Data may be requested from Dr. Siddharth K. Prakash and Dr. Dianna M. Milewicz.
